# Population Genetic Analysis and Sub-Structuring of *Theileria annulata* in Sudan

**DOI:** 10.3389/fgene.2021.742808

**Published:** 2021-11-19

**Authors:** Diaeldin A. Salih, Awadia M. Ali, Moses Njahira, Khalid M. Taha, Mohammed S. Mohammed, Joram M. Mwacharo, Ndila Mbole-Kariuki, Abdelrhim M. El Hussein, Richard Bishop, Robert Skilton

**Affiliations:** ^1^ Biosciences Eastern and Central Africa-International Livestock Research Institute Hub (BecA-ILRI Hub), Nairobi, Kenya; ^2^ Central Veterinary Research Laboratory, Khartoum, Sudan; ^3^ Faculty of Veterinary Medicine, University of Khartoum, Khartoum, Sudan; ^4^ Atbara Veterinary Research Laboratory, Atbara, Sudan; ^5^ Faculty of Veterinary Medicine, University of Al-Butana, Tamboul, Sudan; ^6^ School of Life Sciences, Centre for Genetics and Genomics, University of Nottingham, Nottingham, United Kingdom; ^7^ International Livestock Research Institute, Nairobi, Kenya; ^8^ Central Laboratory, Khartoum, Sudan

**Keywords:** cattle, cell culture vaccine, *Theileria annulata*, genotyping, population genetics, sub-structure, Sudan

## Abstract

*Theileria annulata*, which causes tropical theileriosis, is a major impediment to improving cattle production in Sudan. Tropical theileriosis disease is prevalent in the north and central regions of Sudan. Outbreaks of the disease have been observed outside the known endemic areas, in east and west regions of the country, due to changes in tick vector distribution and animal movement. A live schizont attenuated vaccination based on tissue culture technology has been developed to control the disease. The parasite in the field as well as the vaccine strain need to be genotyped before the vaccinations are practiced, in order to be able to monitor any breakthrough or breakdown, if any, after the deployment of the vaccine in the field. Nine microsatellite markers were used to genotype 246 field samples positive for *T. annulata* DNA and the vaccine strain. North and central populations have a higher multiplicity of infection than east and west populations. The examination of principal components showed two sub-structures with a mix of all four populations in both clusters and the vaccine strain used being aligned with left-lower cluster. Only the north population was in linkage equilibrium, while the other populations were in linkage disequilibrium, and linkage equilibrium was found when all samples were regarded as single population. The genetic identity of the vaccine and field samples was 0.62 with the north population and 0.39 with west population. Overall, genetic investigations of four *T. annulata* populations in Sudan revealed substantial intermixing, with only two groups exhibiting regional origin independence. In the four geographically distant regions analyzed, there was a high level of genetic variation within each population. The findings show that the live schizont attenuated vaccine, Atbara strain may be acceptable for use in all Sudanese regions where tropical theileriosis occurs.

## Introduction

Tropical theileriosis is a tick-borne disease, caused by *Theileria annulata* that continues to be a major concern for livestock in tropical countries affecting millions of animals, particularly crossbreed and exotic cattle, and resulting in significant economic loss and mortality (Dolan, 1989). *Hyalomma anatolicum* transmits *T. annulata* sporozoites causing a lymphoproliferative disease (Ghosh and Azhahianambi, 2007). The sporozoites develop into schizonts which reside inside the host’s lymphocyte and macrophages (Sager et al., 1998). The schizont stage is the only symptomatic stage among different parasite stages in the host, on which attenuated schizont vaccine was designed (Pipano and Shkap, 2006). Many countries, including, Israel (Pipano et al., 1981), Iran (Hashemi-Fesharki, 1988), Russia (Stepanova and Zablotskii, 1989), India (Beniwal et al., 1997), China (Zhang, 1997), Turkey (Sayin et al., 1997), Spain (Viseras et al., 1997), Morocco (Ouhelli et al., 1997), Tunisia (Darghouth, 2008), and India (Roy et al., 2019), have employed the attenuated schizont vaccines to control *T. annulata* infection.

The importance of genetic diversity research in providing information on protozoan parasites, such as epidemiology, control, evolution, virulence, antigenicity, infectivity, treatment sensitivity, and host preference, has been demonstrated (Weir, et al., 2011; Sivakumar et al., 2014). *T. annulata* from other endemic regions, such as China, Oman, Turkey, Tunisia, and Portugal have all been researched utilizing a multilocus genotyping technique for assessing genetic diversity, population structure, and transmission patterns (Weir et al., 2007, 2011; Al-Hamidhi et al., 2015; Gomes et al., 2016; Yin et al., 2018; Roy et al., 2021). *T. annulata* genetic populations studies were notable for its genetic variation, the availability of many genotypes per sample, and sub-structuring by geography (Weir et al., 2007, 2011; Al-Hamidhi et al., 2015; Gomes et al., 2016; Yin et al., 2018; Roy et al., 2019).

Microsatellite-based genotyping was utilized in this work to better understand genetic diversity, population structure, and geographical sub structuring of the *T. annulata* vaccine and parasite samples collected from four different regions in Sudan. The diversity of the markers used would reflect the genetic makeup of the samples as well as the vaccine genetic makeup. The recognition of the vaccine strain would be as fast and efficient if the genetic makeup of the vaccine is the same as the field strain. The findings offer the first glimpse of the *T. annulata* parasites population genetics and diversity in Sudan.

## Materials and Methods

### Cattle Blood Samples

A total of 530 blood samples were collected from cattle in four Sudanese regions using FTA™ cards (Whatman Biosciences, United Kingdom). The four regions were north (*n* = 69), central (*n* = 195), east (*n* = 158), and west (*n* = 108). North and central regions were designated endemic region, while east and west were designated new extension regions. Information on the sampling locations, whether from endemic or new extension regions, total number examined and *T. annulata* positive samples by PCR are provided in Table 1.

**TABLE 1:** Information on sample numbers and location.

Status of the disease	Region	Town	Total number examined	*T. annulata* positive (PCR)
Endemic areas	North Sudan	Atbara field samples	25	14
		Research station	44	22
	Sub total		69	36
	Central Sudan	Khartoum	100	59
		Omderman	79	56
		Madani	4	1
		Singa	9	2
		Kuku	3	2
Sub total			195	120
Grand total			264	156
New extended areas	East Sudan	Kassala	25	22
		Halfa	133	25
	Sub total		158	47
	West Sudan	Nyala	5	4
		Nihod	7	2
		Fashir	6	0
		Obied	90	37
	Sub total		108	43
Grand total			266	90
Grand total			530	246

Out group controls: *Theileria annulata* Ankara strain (Turkey), Tissue culture vaccine strain.

### Extraction of DNA and Small Subunit “SSU” rRNA PCR

Extraction of DNA from cattle blood samples and Atbara vaccine strain was carried out using the PureLink™ Genomic DNA Mini extraction kit (Invitrogen, Germany). For diagnosis of *T. annulata*, the primer used was SSU rRNA gene 989 5′AGT​TTC​TGA​CCT​ATC​AG3′ and the reverse primer was 1,347 5′TGCACAGACCCCAGAG G 3′ giving an amplicon of 370 bp (Allsopp et al., 1993; Taha et al., 2013).

### Microsatellite PCR Assay

In this study, the primers used were designed by Weir et al. (2007). For detection in capillary electrophoresis, the forward primer was labeled with standard labeling dyes at the 5′ end (Supplementary Table S1). The PCR amplification was carried out as described in Salih et al. (2018). For negative control, the nuclease free water was used, while DNA extracted from a schizont-infected lymphocyte culture derived from *T. annulata* Ankara strain was used as a positive control.

### Capillary Electrophoresis and Genotyping

The ABI 3730 Genetic Analyzer (Applied Biosystems-USA) was used to analyze the PCR amplicons at the BecA-ILRI Hub, SegoliP sequencing unit, Nairobi, Kenya. For size fractionation, the Gene Scan 500 LIZ internal lane size standard (Applied Biosystems-USA) was employed. The Gene Mapper tool (Applied Biosystems-USA) was used to score the results, which allowed for the resolution of 1 base pair (bp) changes with many products from a single PCR reaction. The predominant allele was determined as the one with the biggest area under the curve, and amplicons with highest peak height were scored. Allelobin software (Idury and Cardon, 1997) was used to re-sized all Gene Mapper data based on consensus sequence repeats of each marker (Table 2). The inaugural form of file, designated multi locus genotype (MLG) consisted of genotypes created from only the predominant allele at each locus (Weir et al., 2007). The allelic profile dataset, on the other hand, contained genotypic profiles derived from all alleles observed at each locus (where minor peaks were greater than 33% the height of the predominant allele present). The MLG file was used to determine population genetic diversity and structure, while the allelic profile file was utilized to calculate the multiplicity of infection (MOI), as well as to rule out linkage disequilibrium as a null hypothesis.

**TABLE 2:** Major allele frequency, gene diversity, number of alleles and polymorphic information content (PIC) of the nine microsatellite markers used in this study.

Marker	Major allele frequency	Number of allele	Gene diversity (*He*)	PIC
TS5	0.62	10.00	0.58	0.55
TS6	0.46	14.00	0.75	0.73
TS8	0.29	22.00	0.87	0.87
TS9	0.65	4.00	0.46	0.36
TS12	0.48	9.00	0.62	0.54
TS15	0.34	10.00	0.80	0.78
TS20	0.49	18.00	0.67	0.62
TS25	0.25	10.00	0.84	0.82
TS31	0.54	15.00	0.64	0.65
Mean	0.46	12.44	0.70	0.66

### Analyses of Population Genetic

Arlequin v. 3.5 http://cmpg.unibe.ch/software/arlequin 35/ (Excoffier, and Lischer, 2010) was used to calculate the expected heterozygosity, as *Theileria* is haploid and heterozygosity cannot be observed directly. To investigate the genetic relationships between population, principal component analysis (PCA) was calculated in GenAlEx6.5 (Peakall and Smouse, 2006; 2012). Analysis of molecular variance (AMOVA) was performed using ARLEQUIN to test for hierarchical population structure. Nei’s genetic distance (*D*) (Nei, 1978) was calculate between each group of samples from different populations and the vaccine strain using the genetic data analysis tool (GDA) (http://lewis.eeb.uconn.edu/lewishome/gda.html).

The standardized index of association (*I* ^*S*^_*A*_) between each group of samples was estimated using the LIAN 3.7 program, as well as, the degree of linkage disequilibrium (LD) within and between populations (Haubold and Hudson, 2000). After each population was studied separately, the samples were pooled and processed as a single dataset.

STRUCTURE 2.3.4 (http://pritchardlab.stanford.edu/structure.html) was used to investigate population structure employing Bayesian clustering analysis with sample sites as a basis and the admixture scenario with linked allele polymorphism (Pritchard et al., 2000; Evanno et al., 2005). Initial runs of one million steps were used to investigate the datasets (burn-in of 20%). For every value of *K* scale from one (considering all are *T. annulata*) to five (assuming all the five populations are genetically distinct), triplicates were performed. To identify which *K* produced the greatest representation of the data, STRUCTUREHARVESTER 0.6.1 (Earl and von Holdt, 2012) was employed. CLUMPP 1.1 (Jakobsson and Rosenberg, 2007) and DISTRUCT 1.1.2 (Rosenberg, 2004) were used to parse and format the data in order to assess the STRUCTURE output. CLUMPP 1.1 aligns cluster assignments across duplicate analyses, while DISTRUCT 1.1.2 assists with visual representation.

### Multiplicity of Infection

MOI was considered as “existence of numerous genotypes per isolate” when more than one allele was detected at a locus and the smaller peaks were exceed 33% of the height of the predominant allele expressed (Weir et al., 2011; Salih et al., 2018). The mean number of alleles across all nine loci was determined for every sample, and this number was used to indicate the multiplicity of infection in that sample. The multiplicity of infection for each population was calculated by taking the overall mean for each sample’s index value.

## Results

### Verification of Positive Samples for *T. annulata* DNA

**FIGURE 1:**
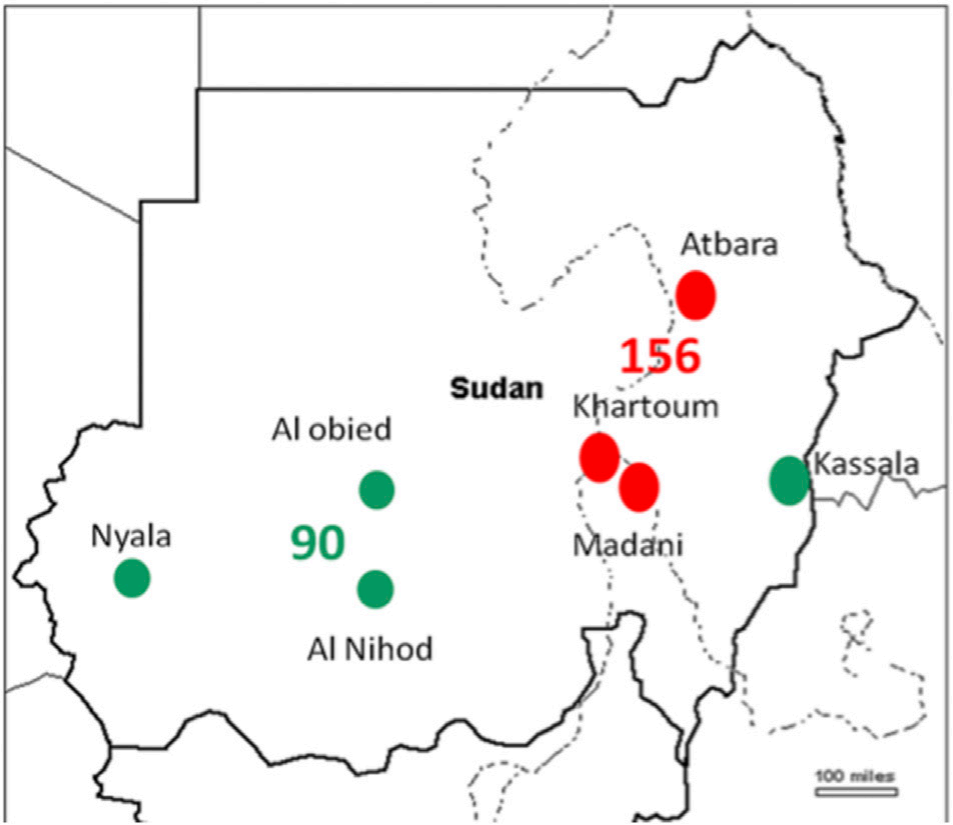
The regions of Sudan where samples were obtained are depicted on the map, the red dots indicates endemic region, while the green ones indicates new extended regions.

*T. annulata* DNA was tested in 530 cattle blood samples. The SSU rRNA PCR assay verified 246 (46.4%) samples positive for *T. annulata* DNA which were subjected to genotyping in addition to the vaccine strain (Table 1). Distribution of the positive samples were as follow, endemic regions *n* = 156 (North *n* = 36, Central *n* = 120) and new extension regions *n* = 90 (East *n* = 47, West *n* = 43) (Figure 1).

### Satellite Marker Diversity

In all of the samples, each marker was highly polymorphic. The polymorphic information content (PIC) of marker TS8 had the highest (0.87), whereas TS9 had the lowest (0.36) (Table 2). This finding argued in favor that these markers could be effective in determining linkage disequilibrium analysis in *T. annulata* populations. The existence of more than one allele at one or more loci confirmed the presence of several genotypes in the samples. For each marker, the number of alleles identified varied from four in TS9 to 22 for TS8 with the mean of 12.44 per marker (Table 2). The dominant allele frequencies varied from 0.25 (TS25) to 0.65 (TS9), with an average of 0.46 (Table 2).

### Population Diversity and Structure

**FIGURE 2:**
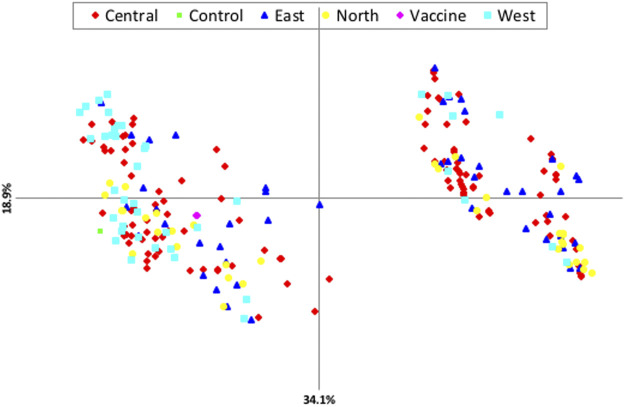
Principle component analysis (PCA) showing the genetic structure of *T. annulata* populations from the four regions of Sudan.

Principal components analysis (PCA) revealed that there is no clustering according to geographical origin (Figure 2). Two sub-structures with a mix of all four populations in both clusters and the vaccine stain being aligned with left-lower cluster were demonstrated, indicating that the parasite populations are rather distinct, with considerable genetic mixing and gene flow between parasites in the four distinct geographical populations investigated.

**FIGURE 3:**
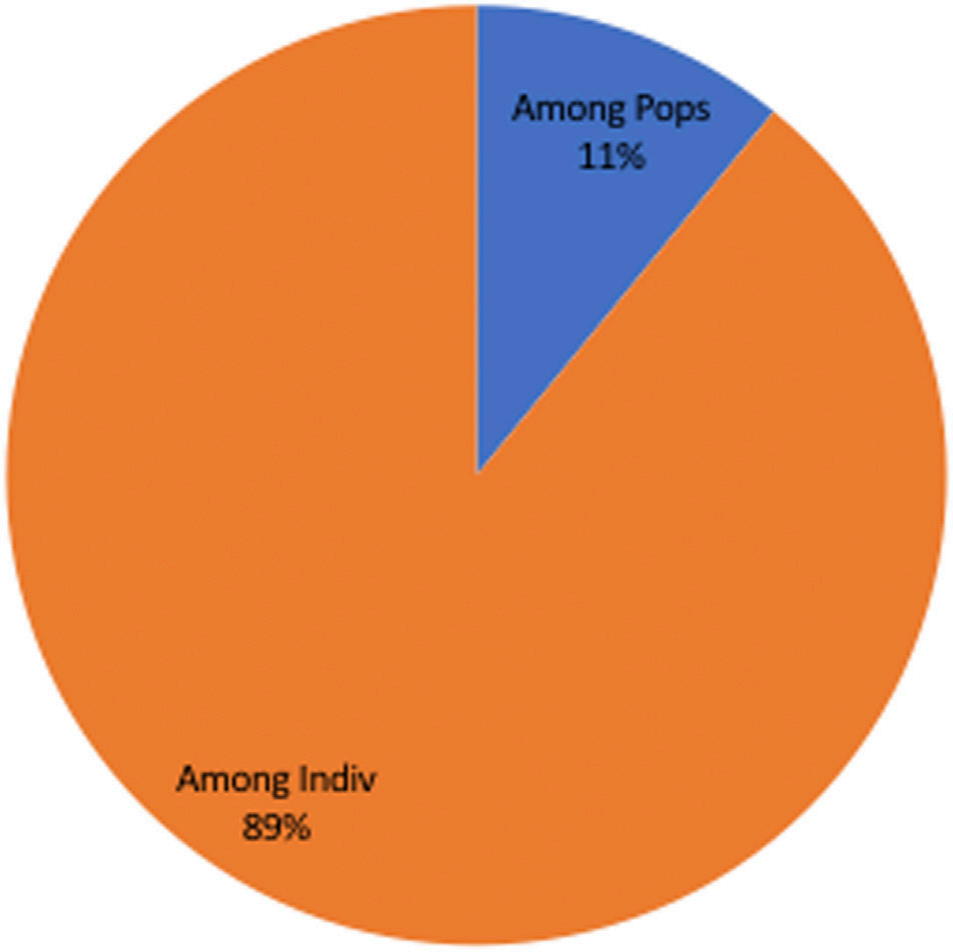
Analysis of Molecular Variation **(**AMOVA) showing only 11% of the genetic variation was explained by populations differences, despite the fact that population differences account for the majority of genetic variety (89%) observed within populations.

The allelic profile data set was examined to see if the *T. annulata* populations observed in Sudan were in linkage equilibrium or disequilibrium. When all the four sub-populations were analysed together (as a single population), the (*I* ^*S*^_*A*_) was positive and greater than zero and the pairwise variance (*V*_D_) was more than the 95% critical value (*L*) suggesting that the merged populations are in linkage equilibrium (LE) (Table 3). The analysis was performed for each population individually to assess for geographic sub-structuring, and three of the populations central, east and west, were shown to be in linkage disequilibrium (LD) (Table 3). Only 11% of the genetic variation was explained by variations between populations, which account for a considerable portion of the genetic diversity (89%) detected within populations (Figure 3).

**TABLE 3:** Linkage equilibrium analyses in Sudanese population of *Theileria annulata*.

Population	*I*_A_^S^	*V*_*D*_	*L*_*para*_	*L*_*MC*_	Linkage
North	0.0311	2.0792	1.9275	1.9992	LE
Central	0.0055	1.9965	2.1102	2.0798	LD
East	−0.0049	1.7881	2.3669	2.5958	LD
West	0.0037	1.9048	2.7354	2.7937	LD
All population	0.0174	2.1006	1.9591	1.9760	LE

*I*_A_^S^, standard index of association; *V*_*D*_, mismatch variance (linkage analysis); LD, linkage disequilibrium; LE = linkage equilibrium; *L*_*MC*_ and *L*_*para*_, upper 95% confidence limits of Monte Carlo simulation and parametric tests respectively (linkage analysis).

Estimating Nei’s genetic distance (*D*) between each of the four regionally sampled populations as well as between them and the vaccine strain, was used to evaluate genetic differentiation between the four populations (Table 4). The genetic differentiation between central and east populations (*D* = 0.82) was greater than that observed between the east and west populations (*D* = 0.64). The population with the lowest genetic distance from the vaccine genotype was west (*D* = 0.39), while the most genetically similar was north (*D* = 0.62) (Table 4).

**TABLE 4:** The Nei genetic destance between the four populations studied and the vaccine strain.

	Central	East	North	Vaccine	West
Central	1.00	—	—	—	—
East	0.82	1.00	—	—	—
North	0.85	0.83	1.00	—	—
Vaccine	0.51	0.53	0.62	1.00	—
West	0.81	0.64	0.70	0.39	1.00

**FIGURE 4:**
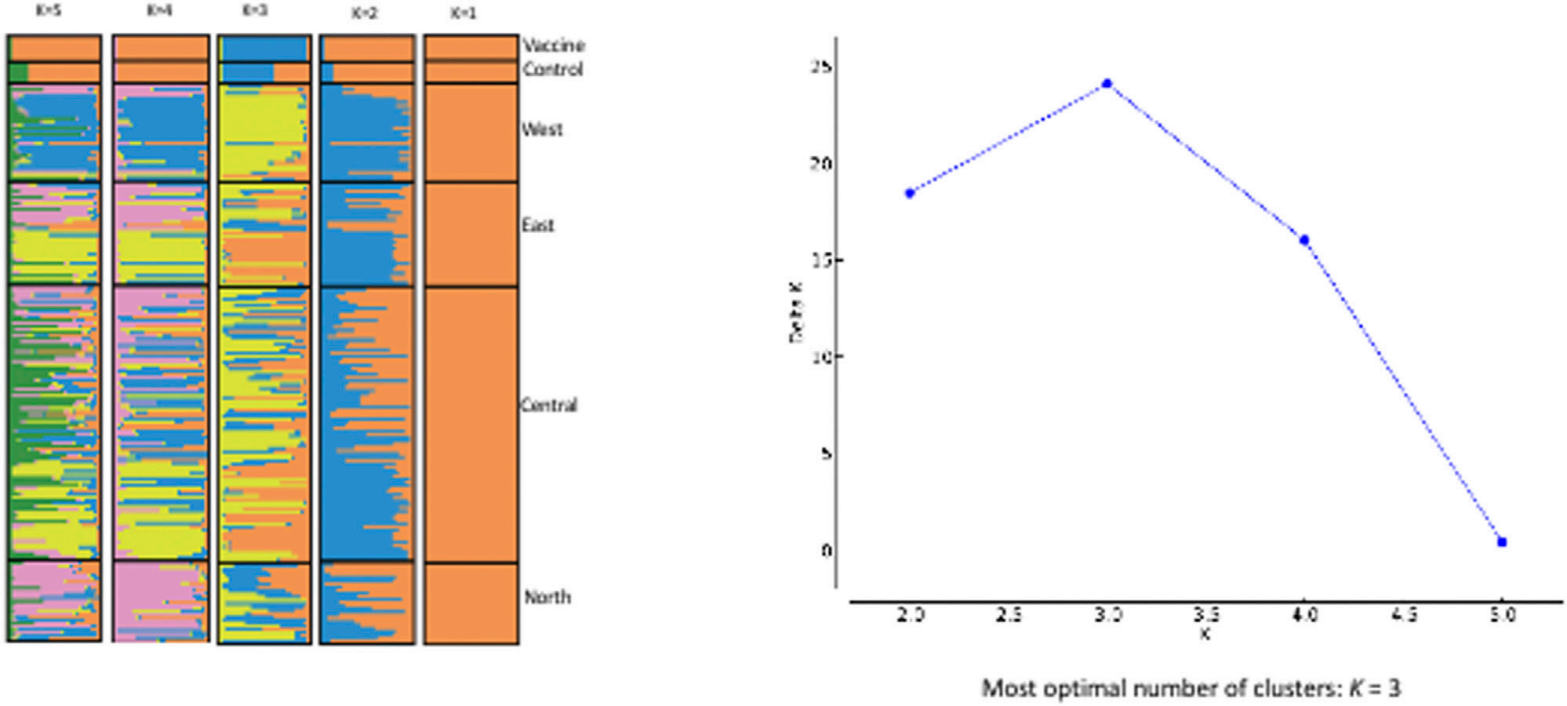
EVANNO Method Delta and STRUCTURE. The graph shows optimal number of clusters from the STRUCTURE analysis; STRUCTURE analysis from K = 2 to K = 5 with samples from the four regions of Sudan.

Based on the Evanno et al. delta K technique, the STRUCTURE results imply that *K* = 3 is the optimal number of genetic groups to define the genotypes of Sudanese *T. annulata* populations as well as in *T. annulata* vaccine strain (Figure 4). The three clusters are designated as gene pool 1, 2 and 3 respectively. Gene pool 1 (purple colour) prevailed in central and east, while gene pool 2 (blue colour) were most prevalent in north and vaccine, and pool 3 (yellow colour) predominated in west (Figure 4). In the vaccine strain, gene pool 2 appears to be more common than gene pool 1.

### Multiplicity of Infection

Multiple genotypes were observed in *T. annulata* populations from the four geographic regions, with multiple alleles being found at one or more loci. The mean number of alleles for the nine loci was determined for each sample, to obtain an index value that denoted multiplicity of infection. Table 5 summarizes the multiplicity of infection for each population and across all four populations analyzed. North and central populations had high mean of values of 1.75 and 1.59, respectively, while east and west had values of 1.33 and 1.03, respectively.

**TABLE 5:** Multiplicity of infection in Sudanese *Theileria annulata* population.

Population	*n*	Multiplicity of infection			
		Mean	SD	Min	Max
North	36	1.75	0.90	0.56	3.22
Central	120	1.59	0.83	0.69	2.91
East	47	1.33	0.88	0.45	2.89
West	43	1.03	0.75	0.17	2.43
All	246	1.43	0.84	0.47	2.86

*n*, number of samples and SD, standard deviation.

## Discussion

Using microsatellite markers, this study investigated the diversity and population structure of *T. annulata* in Sudan. The study’s samples (*n* = 246) were obtained from four different geographical regions. North and central regions known to be endemic of *T. annulata* since the eighties, and the remaining two (west and east) witnessed the spreading of the disease in the nineties. In addition, the *T. annulata* vaccine from Sudan was also included in the study. In order to gain insight into the epidemiology of a parasite, ascertain sources of infection and modes of transmission, it is critical to assess population, genetic diversity and structure (Weir et al., 2007, 2011; Salih et al., 2018).

The genetic diversity and population structure of *T. annulata* found in Sudan were studied using a panel of nine microsatellite markers. The highest mean genetic diversity was observed in north, a finding which could be due to significant tick infestations in this region, where the disease has been established for long time (El Hussein, et al., 2012; Gharbi et al., 2020). The lower degree of *T. annulata* diversity detected in the parasite population from the west corresponded to the recent reported of tropical theileriosis (Mohammed-Ahmed et al., 2020). In other countries where tropical theileriosis is endemic, a comparable scale of genetic variation has been observed among *T. annulata* populations (Weir et al., 2011; Al-Hamidhi et al., 2015; Gomes et al., 2016; Yin et al., 2018; Roy et al., 2021).

The results revealed relatively slight geographical sub-structuring among the four populations of *T. annulata* in Sudan with no evidence of grouping based on geographical origin. The fact that resources (feeds and water) are collectively utilized under the nomadic cattle systems prominent in Sudan is essential to enhance genetic uniformity. This result is supported by PCA analysis as well as STRUCRURE results. AMOVA revealed a high percentage of crossing between various *T. annulata* samples as well as recombination within the parasite population. Individual samples, rather than groups derived from a specific geographic region, accounted for the majority of genetic variation. In the future, other aspects such as parasite challenge and quantifying the extent of tick infestation should be examined. PCA and AMOVA results figured out no evident link between population genetic structure and the geographical origin of the isolates investigated. However, PCA analysis revealed a close genetic link between the north and *T. annulata* vaccine genotypes, with the *T. annulata* vaccine and majority of north genotypes clustered together.

When the PCA and STRUCTURE data are combined, it can be expected that there are three potential populations of *T. annulata* in Sudan. It’s possible that gene pool 1 is introgressing into gene pool 2 or vice versa, with the two gene pools will eventually merging into one. This conclusion could be a result of cattle migration being unfettered across the country, due to the lack of trade barriers and policies restricting livestock movement (Oura et al., 2005; Roy et al., 2019). The mobility of parasite-infected/tick-infested cattle from one region to another assists in population homogenization.

The extent of linkage equilibrium between alleles at pairs of loci was evaluated, to see if the *T. annulata* populations in the four regions of Sudan constituted a single panmictic population with a high degree of genetic exchange. When the samples from the four regions were analyzed as a single population, an *I* ^*S*^_*A*_ value of 0.0174 was obtained as well as a *V*_D_ value (2.1006) that was greater than L (1.9591), demonstrating LE. The presence of LE in the combined populations could be due to an epidemic population structure (Smith et al., 1993), or it could be due to occasional genetic exchange, resulting in a clonal population structure (Wier et al., 2011). Other factors that could contribute to the reported LE include inbreeding, recombination rate and the size of the regional parasite functional population (Charlesworth, 2009). More samples from Sudan are needed to clarify which characteristics are most essential, especially because a limited number of genetically identical parasites in the vertebrate host could result in substantial linkage disequilibrium (Anderson et al., 2000).

The highest level of multiplicity of infection (MOI) was identified in the north, with a highest value of 3.22, followed by east, with lowest and maximum values of 0.45 and 2.89, respectively showing a significant degree of variability in the dataset. In the midgut of the tick vector, multiple infections stimulate cross-mating and recombination among distinct parasite genotypes, as well as the formation of unique recombinant genotypes (Weir et al., 2001; Al-Hamidhi et al., 2015; Salih et al., 2018). The higher number of *T. annulata* genotypes in north could enable a high rate of cross-mating and recombination, resulting in increased genetic diversity in the bovine host (Conway et al., 1999). It could be also explained by the high tick load reported in north compared to the other regions (Salih et al., 2004).

In conclusion, the application of polymorphic microsatellite loci has offered preliminary insight into the population genetic diversity and structure of *T. annulata* population in Sudan. Extensive genetic intermixing between the four *T. annulata* populations studied was indicated, as well as minimal evidence of genetic differentiation and a high level of genetic diversity within each population. The findings show that the vaccine (Atbara strain) could be used in all areas where tropical theileriosis present.

*T. annulata* populations found in north African countries where tropical theileriosis is currently an economically important disease, should be examined and compared to see how genetically similar they are. Such data can assist veterinary control policy makers in determined if preventative measures, such as immunization, should be deployed at the national, regional or continental level.

## Data Availability

The raw data supporting the conclusion of this article will be made available by the authors, without undue reservation.

## Supplementary Material

Supplementary Table 1 Primers information used.
