# Supplementary material for: Population Genetic Analysis and Sub-Structuring of Theileria annulata in Sudan
Source: Front Genet. 2021 Nov 19;12:742808. doi: 10.3389/fgene.2021.742808 (PMC8640526; doi:10.3389/fgene.2021.742808)
Supplement: Supplementary file 1 [file Table1.docx]

Table S1: Primers information used

| **Chrmsm.** | **Name** | **Sequence** | **Bases** | **Dye** | **Size range of alleles (based on Weir et al. 2007)** | **Consensus repeat sequence** | **Copy number of repeats** |
| --- | --- | --- | --- | --- | --- | --- | --- |
| 4 | TS5-F | ctggaacatgaattacttgttcttcc | 26 | ned | 240–318 | GGTTCA | 13.8 |
|  | TS5-R | ggacaccaatgagtgacgtgacag | 24 |  |  |  |  |
| 4 | TS6-F | catcctttgacctactgattgtac | 24 | 6-fam | 301–466 | TAATTATAGG | 13.6 |
|  | TS6-R | cggtagtaccagttaatactgtc | 23 |  |  |  |  |
| 3 | TS8-F | taaacgattaaaatcaagtg | 20 | vic | 195–356 | TATTATTTAATG | 11.2 |
|  | TS8-R | attggaaatggtgaaataatgag | 23 |  |  |  |  |
| 3 | TS9-F | aatgtgtggtacaacatcac | 20 | pet | 338–386 | ATT | 27.3 |
|  | TS9-R | gatatggaatcatactagaagttg | 24 |  |  |  |  |
| 3 | TS12-F | gatgatagaggaattgatatgac | 23 | 6-fam | 237–376 | AATACT | 10 |
|  | TS12-R | ggaaatatcacaattaagattc | 22 |  |  |  |  |
| 1 | TS15-F | gtacgtaatcttggaaatggtag | 23 | vic | 164–404 | AAGATACTAATGGAAGATTAAGTA | 6.7 |
|  | TS15-R | gatacaacgttacggagtcagttgg | 25 |  |  |  |  |
| 2 | TS20-F | ccttcatgatctacatctgatgc | 23 | ned | 187–310 | ATTATTACTA | 11.7 |
|  | TS20-R | ggctgaatgggtacctgttc | 20 |  |  |  |  |
| 4 | TS25-F | cgccatcagtagtcatctcag | 21 | pet | 209–296 | ATTATACTATACTATT | 6.6 |
|  | TS25-R | gacgaccataactgggaagtcaac | 24 |  |  |  |  |
| 2 | TS31-F | gttatcttcttgctattatagc | 22 | ned | 180–340 | ttatatagttaagt | 17.9 |
|  | TS31-R | gtattaaaatctataagattc | 21 |  |  |  |  |
